# Supplementary material for: Brain microvasculature defects and Glut1 deficiency syndrome averted by early repletion of the glucose transporter-1 protein
Source: Nat Commun. 2017 Jan 20;8:14152. doi: 10.1038/ncomms14152 (PMC5263887; doi:10.1038/ncomms14152)
Supplement: Supplementary Information — Supplementary Figures. [file ncomms14152-s1.pdf]

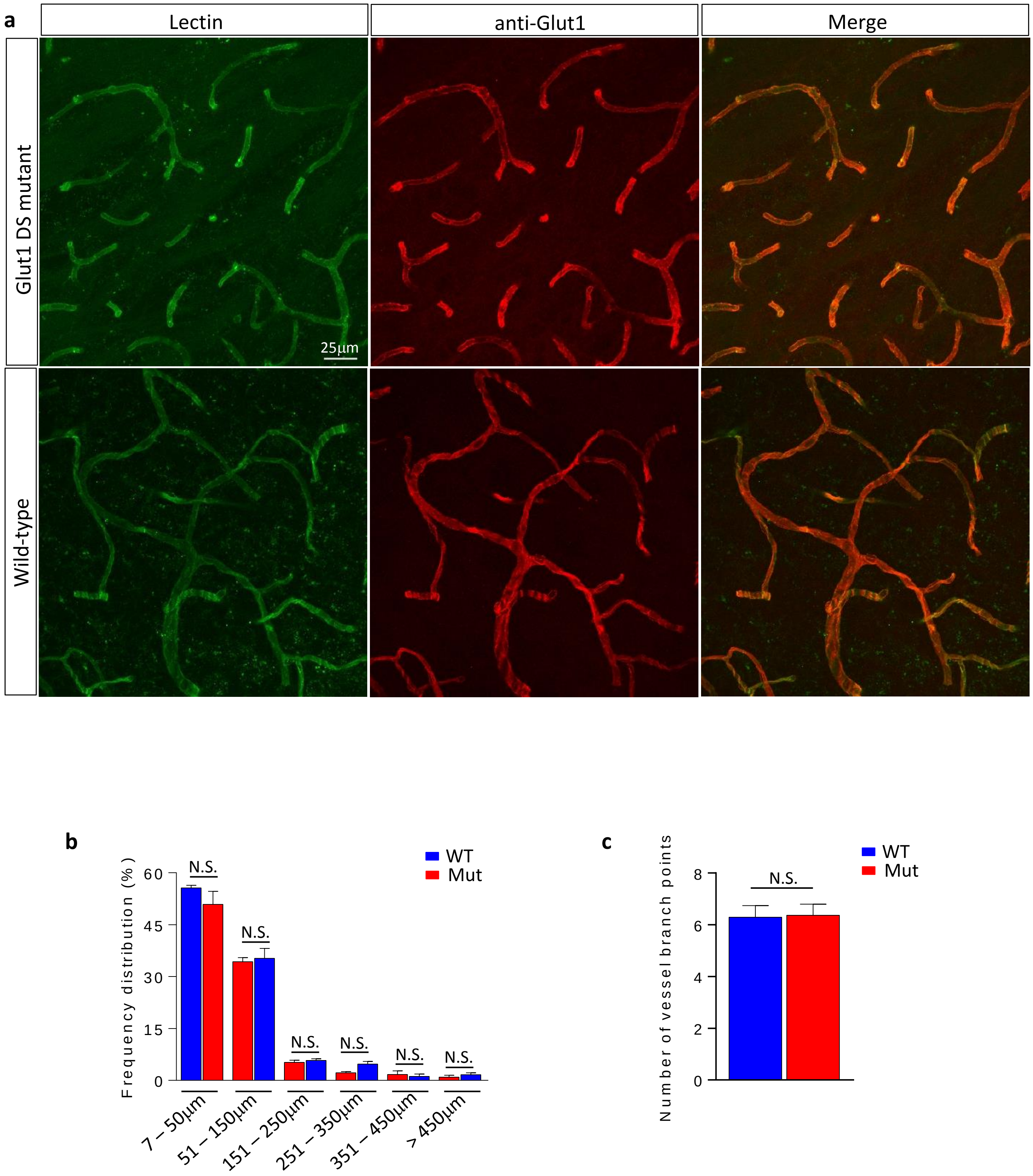

Supplementary Figure 1. **Brain microvasculature of Glut1 DS and WT mice**. **(a)** Immunohistochemistry of brain sections with labeled lectin or an antibody against the Glut1 protein demonstrates perfect alignment of the two probes. Depicted are representative sections of the thalamus from a 6-month old Glut1 DS mutant and WT littermate. A frequency distribution of individual capillaries of various sizes (lengths) **(b)** or an assessment of vessel branch points **(c)** fails to detect differences between the cerebral microvasculature of Glut1 DS mutants and WT littermates at 2 weeks. $P > 0.05$, t test, N ≥ 3 mice of each genotype.

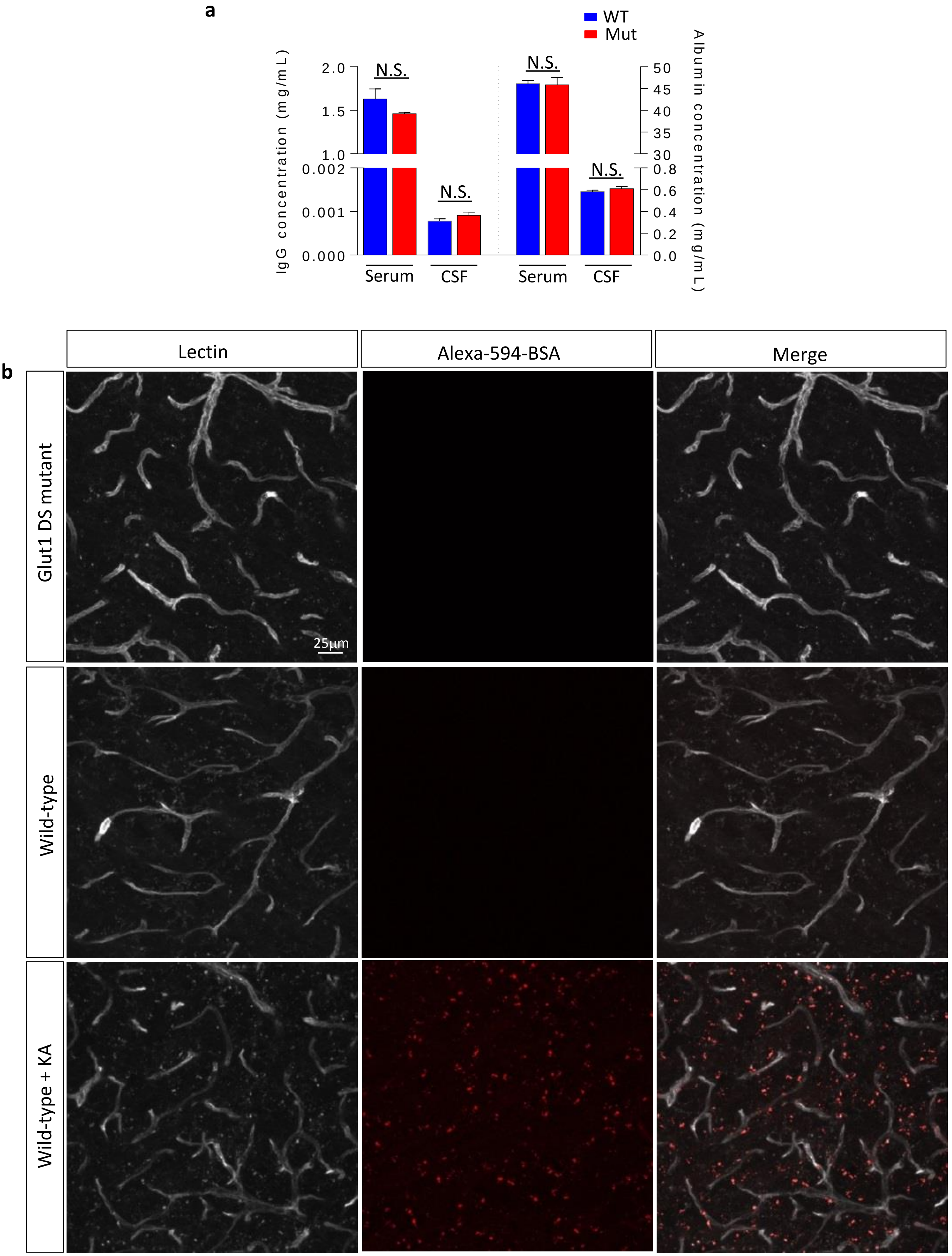

Supplementary Figure 2. The integrity of the blood-brain barrier is preserved in Glut1 DS model mice. (a) CSF and serum concentrations of albumin and IgG remain unchanged in Glut1 DS model mice. $P > 0.05$, *t* test, N ≥ 4 mice of each genotype. **(b)** The BBB of mutant mice remains intact as determined by the absence of fluorescently labeled albumin (BSA) in the brain parenchyma following administration of the probe. Representative thalamic sections are depicted. Note fluorescence in the parenchyma of mice pre-treated with kainic acid.

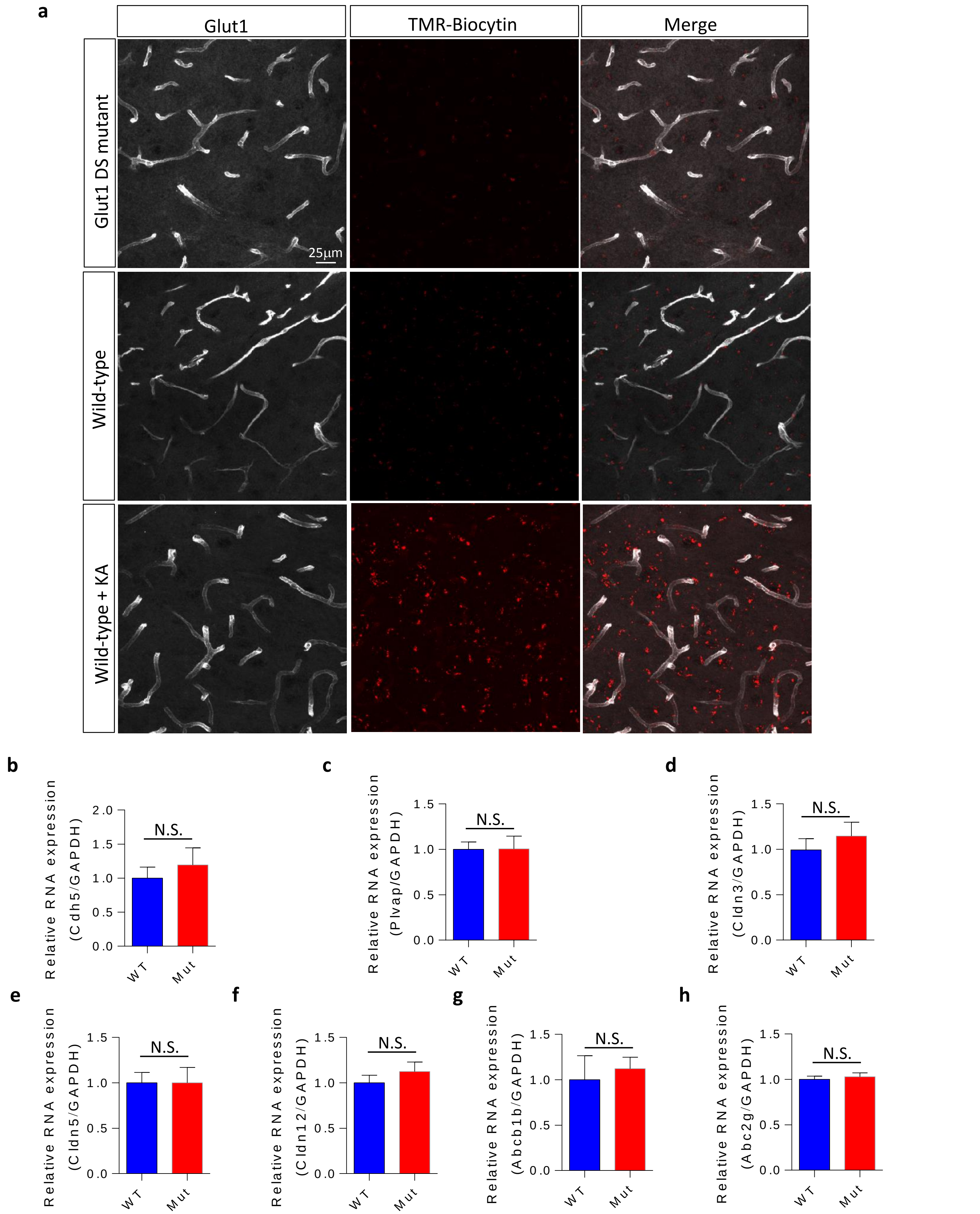

Supplementary Figure 3. The Glut1-deficient blood-brain barrier is impervious to small molecules and fails to exhibit perturbations in BBB signature genes. (a) Immunohistochemistry of sections from the thalamus of mice administered the small-molecule tracer TMR-bioctyin indicates that haploinsufficiency of Glut1 does not compromise the BBB. Note absence of major differences in the intensity of TMR-biocytin in the neuropil of wild-type and mutant mice. Faint TMR-biocytin puncta outside the vessels is considered normal and observed in unaffected mice too (Ref. 22). **(b – h)** Graphs depicting quantitative PCR results of the expression of BBB endothelial "signature" genes in brain tissue of 20-week old Glut1 DS model mice and WT controls. $P > 0.05$, *t* test, N ≥ 6 mice of each genotype.

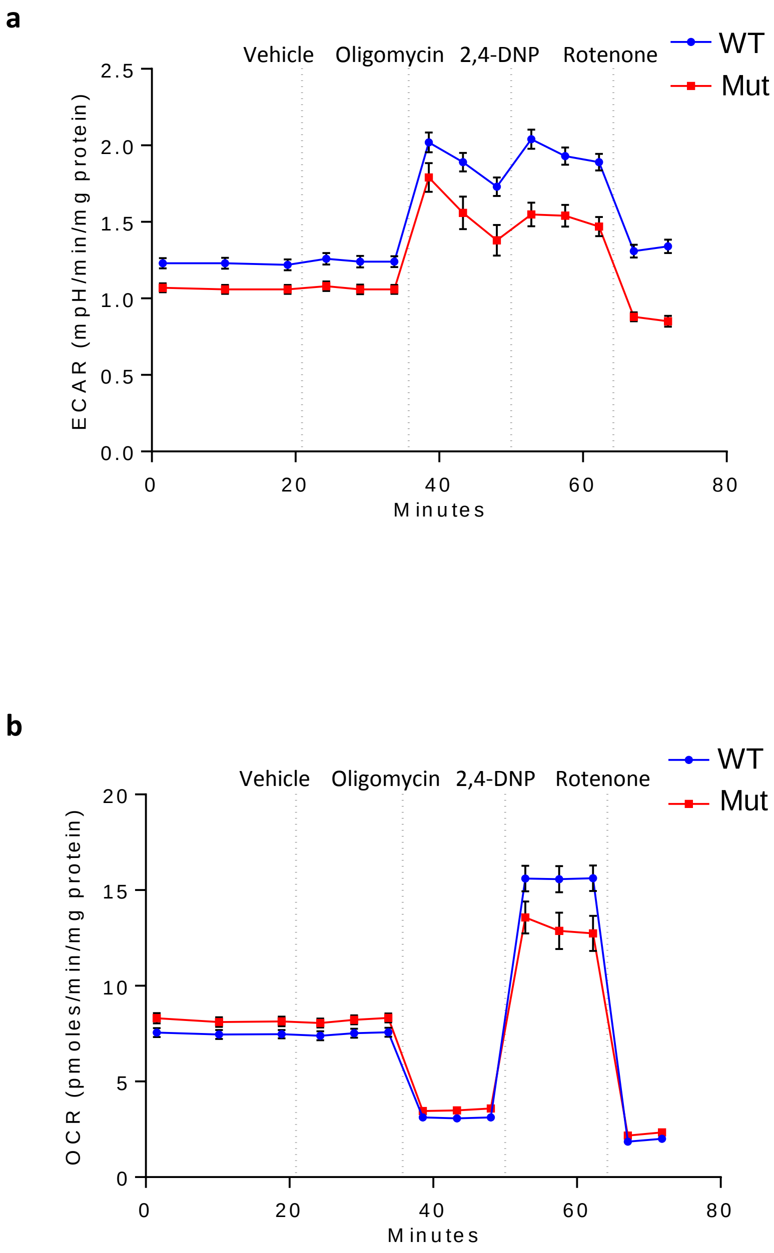

Supplementary Figure 4. Glycolytic flux and maximal respiratory capacity declines in Glut1 DS cells. (a) Glycolytic flux, as assessed by the rate of extra-cellular acidification (ECAR), under basal conditions and following injection of metabolic drugs into the culture medium, is reduced in Glut1 DS cells. **(b)** Mitochondrial respiration, as assessed by oxygen consumption rate (OCR) depicts an inability of the mutant cells to attain the maximal respiratory capacity of wild-type cells – see traces following administration of 2, 4-Dinitrophenol (2, 4-DNP). For quantification of the parameters, refer to Fig. 3.

a

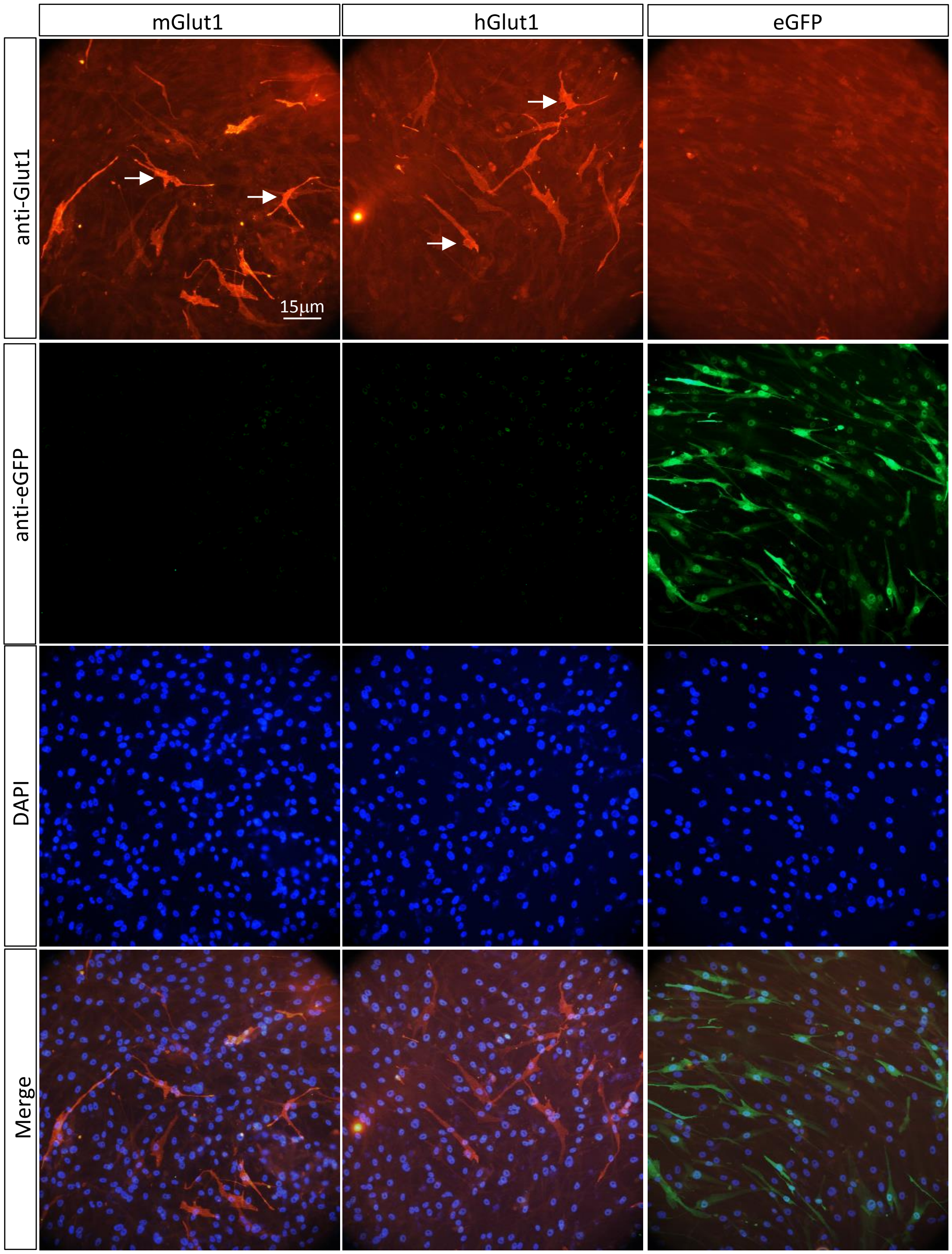

b

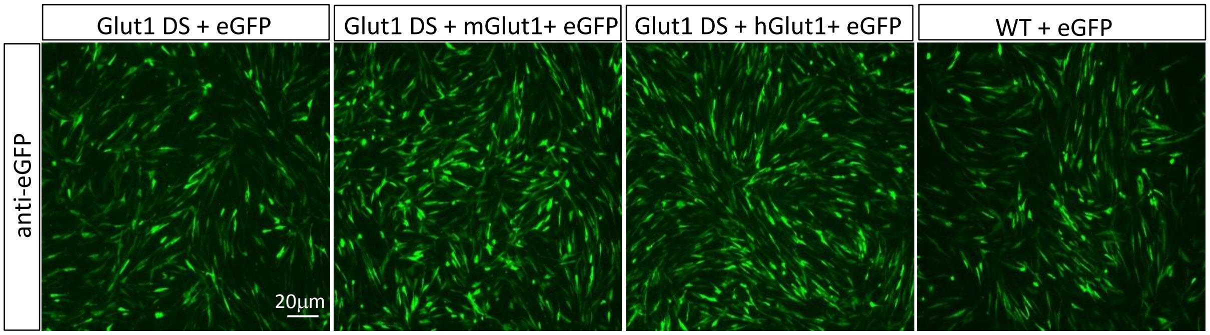

Supplementary Figure 5. **Analysis of Glut1 constructs in cultured cells. (a)** Glut1 DS patient fibroblasts transfected with either a murine or human Glut1 cDNA construct but not an eGFP cassette express high levels of the Glut1 protein (arrows). **(b)** Efficient transduction of Glut1 DS fibroblasts subjected to the glucose uptake assay. Depicted are cells from each of the experiments stained for eGFP which was co-transfected into the fibroblasts along with the Glut1 constructs.

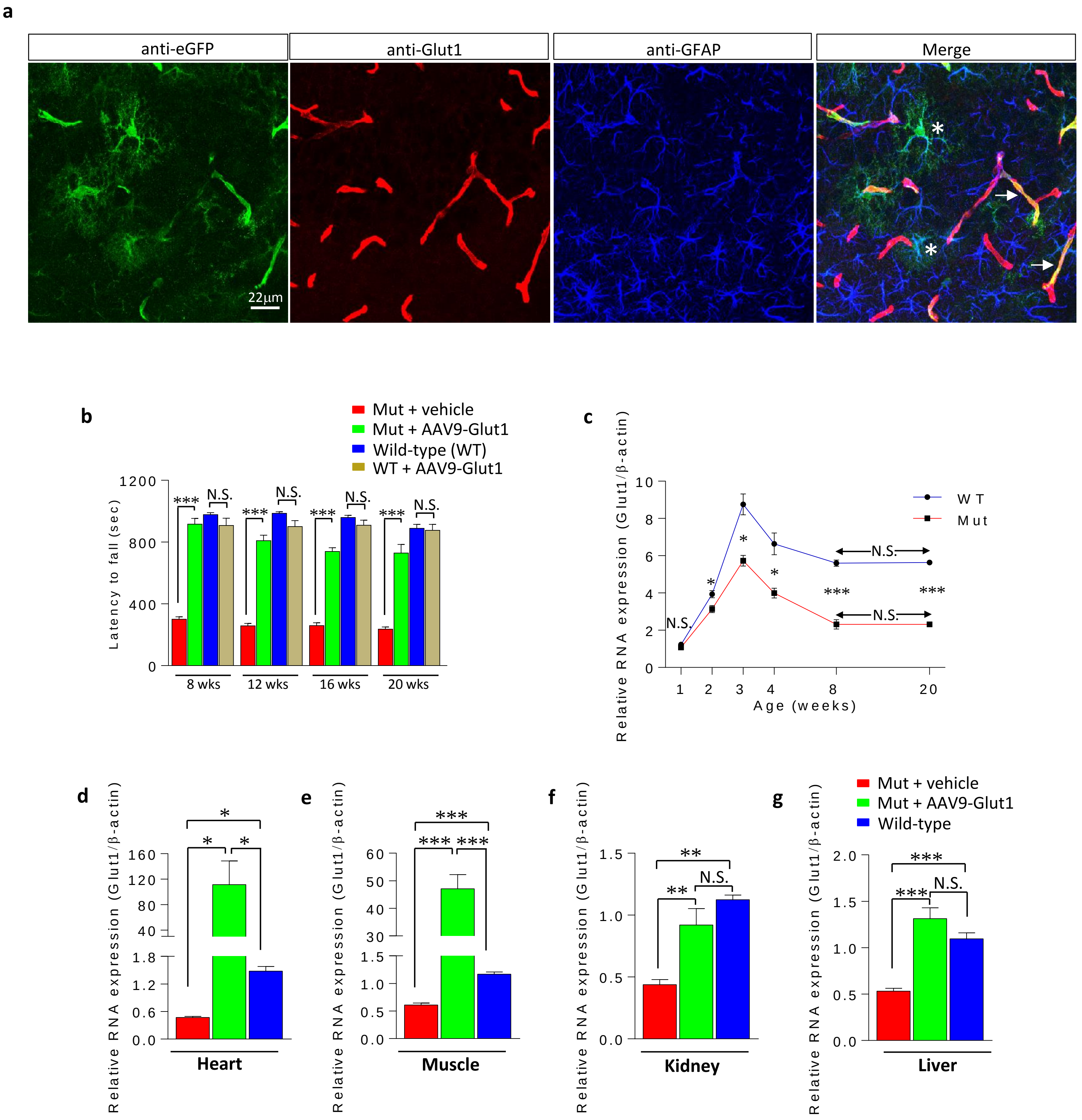

Supplementary Figure. 6. Assessing the delivery and effects of AAV9-Glut1 on Glut1 DS model mice. (a) Immuno-histochemistry of brain (hippocampus) sections of mice administered AAV9-eGFP showing transduction of the Glut1-positive cells of the cerebral microvasculature by the vector (arrows). Note – asterisks denote astrocytes. **(b)** Graphical representation of improved motor performance on the rotarod following administration of the AAV9-Glut1 vector through the intra-cerebral ventricles of PND3 Glut1 DS model mice. Systemic administration of the vector into WT mice did not improve performance above that observed in naïve WT mice. ***, $P < 0.001$, *t* test, N ≥ 7 mice in each cohort. **(c)** Longitudinal assessment of Glut1 mRNA expression in brain tissue of Glut1 DS mutants and control, WT littermates. Expression peaked at 3 weeks of age in both cohorts, leveling off at ~8 weeks. A significant difference between mutants and controls became apparent at 2 weeks. *, ***, $P < 0.05$ and $P < 0.001$ respectively, *t* test, N ≥ 3 mice of each genotype. **(d – g)** Glut1 repletion in mutants at PND3 significantly raises Glut1 RNA expression in diverse organs. *, **, ***, $P < 0.05$, $P < 0.01$ and $P < 0.001$ respectively, one-way ANOVA, N ≥ 3 mice in each cohort.

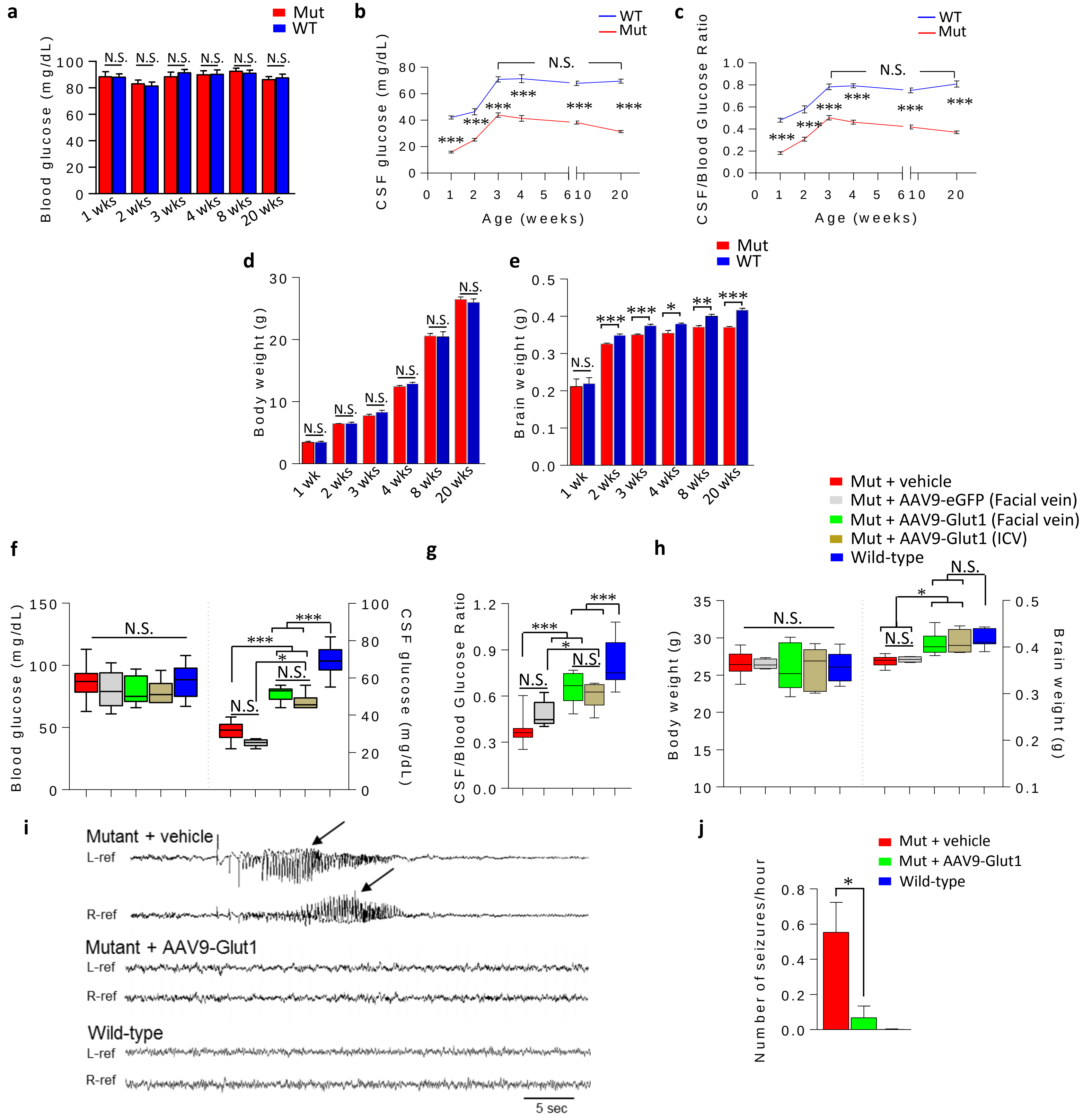

Supplementary Figure 7. **Effects of early Glut1 repletion on the hypoglycorrhachia, micrencephaly and epileptiform activity of Glut1 DS model mice.** Longitudinal assessment of **(a)** blood glucose, **(b)** CSF glucose and **(c)** the ratio of CSC:blood glucose in naïve (untreated) mutants and WT littermates demonstrates unchanged blood glucose values. In contrast, significant differences in CSF glucose values and CSF:blood glucose ratios become evident as early as 1 week of life. Moreover, in mutants both values declined significantly from a peak recorded at 3 weeks of age. ***, $P < 0.001$, *t* test, N ≥ 7 mice of each genotype. **(d)** Body weight of Glut1 DS mutants is no different from that of WT, control littermates between 1 and 20 weeks of age. However, diminished brain size, a measure of micrencephaly becomes evident in mutants **(e)** no later than PND14. *, **, ***, $P < 0.05$, $P < 0.01$, $P < 0.001$ respectively, *t* test, N ≥ 5 mice of each genotype for (D) and (E). AAV9-Glut1 delivered early into the intra-cerebral ventricles is also effective in **(f, g)** mitigating hypoglycorrhachia and **(h)** restoring normal brain size to Glut1 DS model mice. **, ***, $P < 0.01$ and $P < 0.001$ respectively, one-way ANOVA, N ≥ 8 mice in each group. Results of EEG analysis depicting **(i)** representative brain-wave activity in the three cohorts of mice and **(j)** a quantification of epileptic seizure-like events. *, $P < 0.05$, one-way ANOVA, N ≥ 3 mice in each group. Note persistence of spontaneous, generalized seizures (arrow) in the vehicle-treated animal.

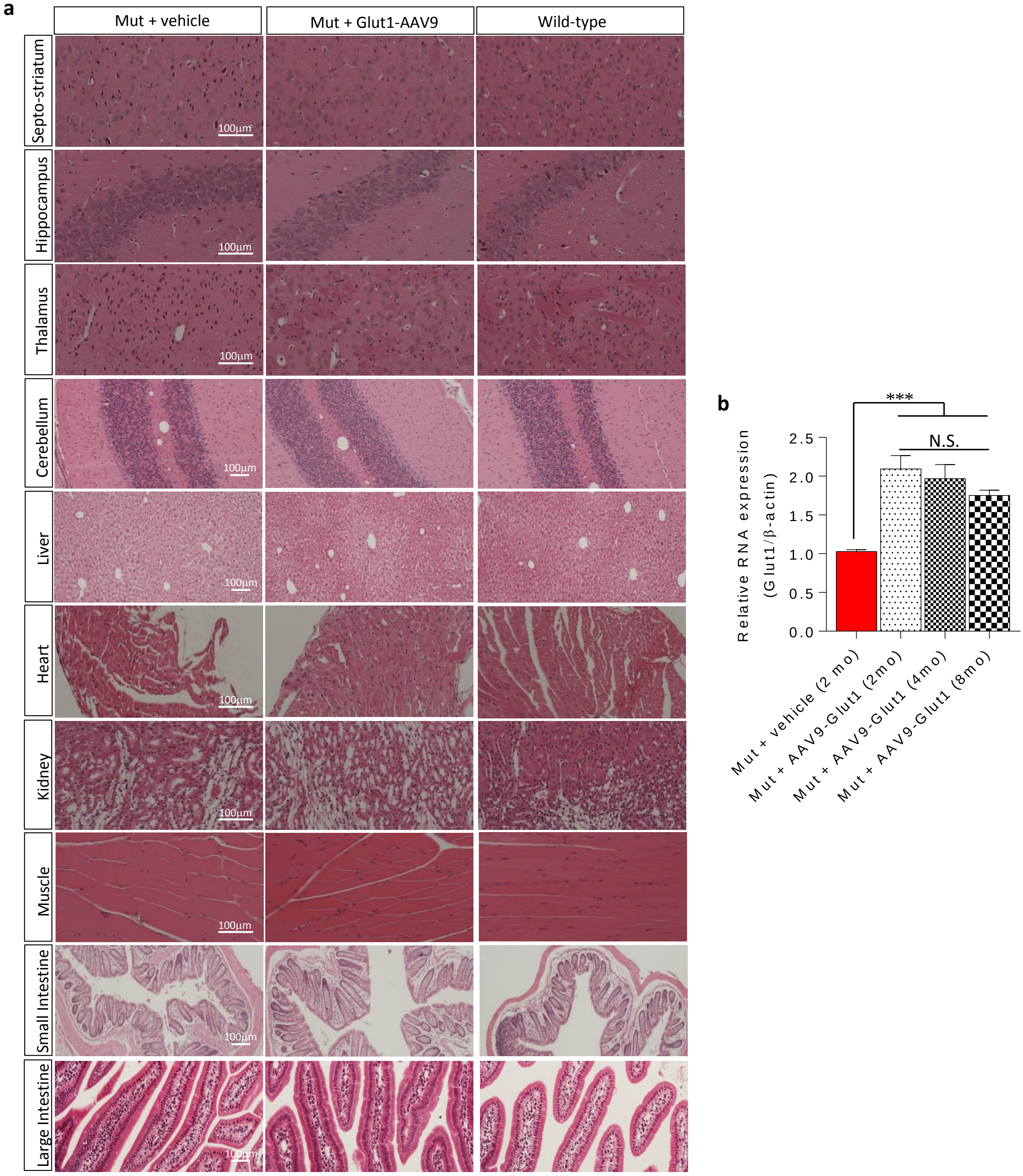

Supplementary Figure 8. Early administration of AAV9-Glut1 allows for sustained expression of the Glut1 gene and does not trigger major pathology in treated mice. **(a)** Hematoxylin/eosin histochemistry of the major organs of the body at 5 months of age depicts normal cellular morphology in the majority of the tissues of mutants treated with AAV9-Glut1. A hint of steatosis within the liver tissue is detected; note somewhat lighter staining of tissue (asterisk) surrounding central vein in the sample from the mutant treated with the Glut1 virus. **(b)** QPCR analysis of Glut1 transcripts in brain tissue reveals sustained expression of the Glut1 transgene as late as 8 months of age. Note significant increase in expression relative to that in the vehicle-treated mutant. ***, *P* < 0.001, one-way ANOVA, N ≥ 3 mice in each cohort.

a

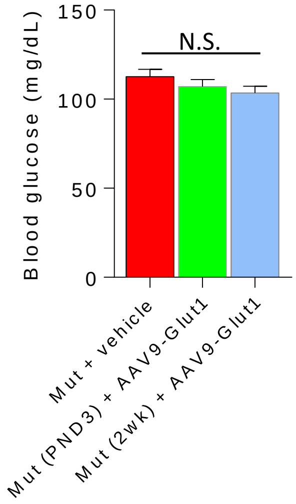

Supplementary Figure 9. **Blood glucose levels in mutants provided food *ad libitum***. Blood glucose levels remain unaltered in non-fasted 5-month old mutants administered AAV9-Glut1. $P > 0.05$, one-way ANOVA, N = 3 – 8 mice in each cohort.

Full-sized western blots

Fig. 3b

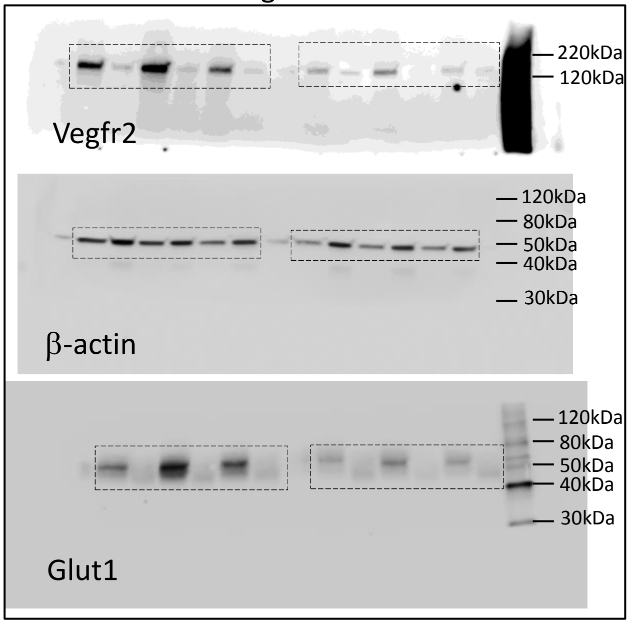

Fig. 3e

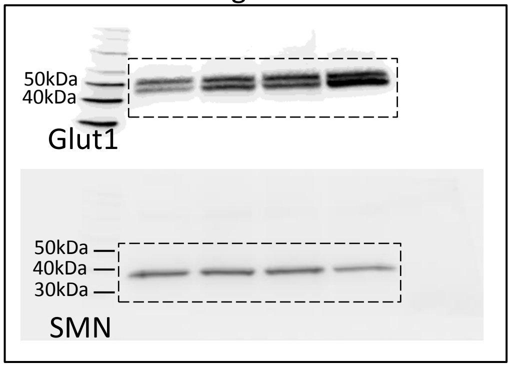

Fig. 4d – upper panel

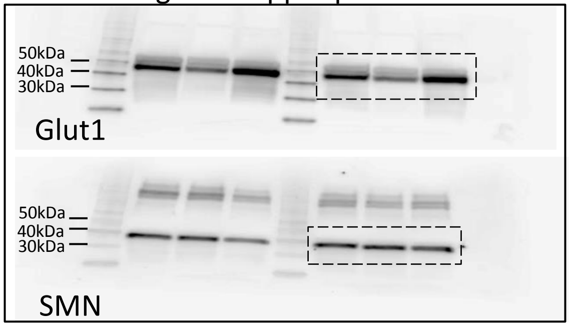

Fig. 4d – lower panel

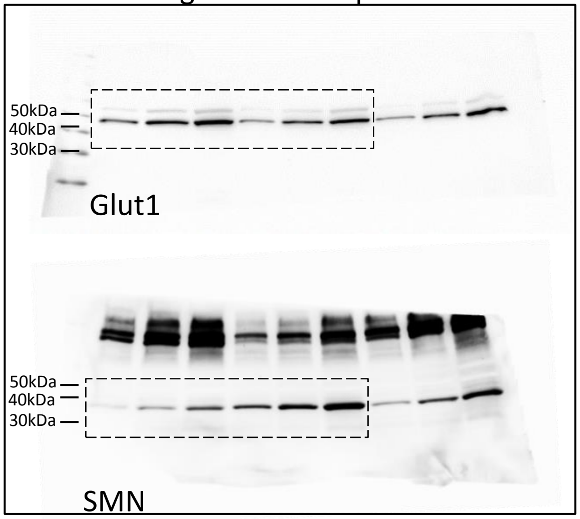

Fig. 6c

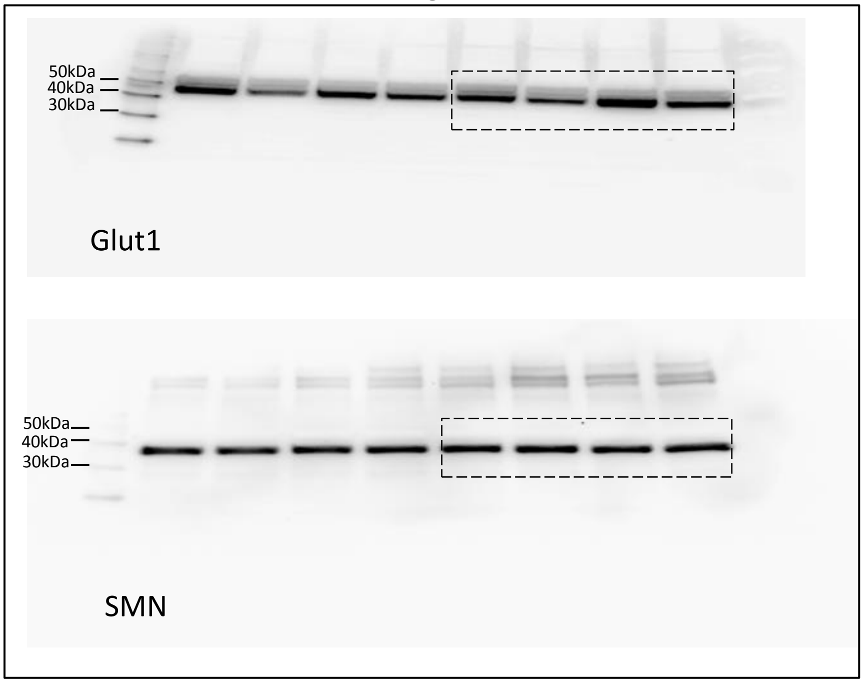

Supplementary Figure 10. Images of full-sized western blots.
